# Beat-to-Beat Vectorcardiographic Analysis of Ventricular Depolarization and Repolarization in Myocardial Infarction

**DOI:** 10.1371/journal.pone.0049489

**Published:** 2012-11-14

**Authors:** Muhammad A. Hasan, Derek Abbott, Mathias Baumert

**Affiliations:** 1 School of Electrical & Electronic Engineering, The University of Adelaide, Adelaide, Australia; 2 Centre for Heart Rhythm Disorders, The University of Adelaide, Adelaide, Australia; 3 Centre for Biomedical Engineering (CBME), The University of Adelaide, Adelaide, Australia; Albert Einstein College of Medicine, United States of America

## Abstract

**Objectives:**

Increased beat-to-beat variability in the QT interval has been associated with heart disease and mortality. The purpose of this study was to investigate the beat-to-beat spatial and temporal variations of ventricular depolarization and repolarization in vectorcardiogram (VCG) for characterising myocardial infarction (MI) patients.

**Methods:**

Standard 12-lead ECGs of 84 MI patients (22 f, 63±12 yrs; 62 m, 56±10 yrs) and 69 healthy subjects (17 f, 42±18 yrs; 52 m, 40±13 yrs) were investigated. To extract the beat-to-beat QT intervals, a template-matching algorithm and the singular value decomposition method have been applied to synthesise the ECG data to VCG. Spatial and temporal variations in the QRS complex and T-wave loops were studied by investigating several descriptors (point-to-point distance variability, mean loop length, T-wave morphology dispersion, percentage of loop area, total cosine R-to-T).

**Results:**

Point-to-point distance variability of QRS and T-loops (0.13±0.04 vs. 0.10±0.04, *p*< 0.0001 and 0.16±0.07 vs. 0.13±0.06, *p*< 0.05) were significantly larger in the MI group than in the control group. The average T-wave morphology dispersion was significantly higher in the MI group than in the control group (62°±8° vs. 38°±16°, *p*< 0.0001). Further, its beat-to-beat variability appeared significantly lower in the MI group than in the control group (12°±5° vs. 15°±6°, *p*< 0.005). Moreover, the average percentage of the T-loop area was found significantly lower in the MI group than the controls (46±17 vs. 55±15, *p*< 0.001). Finally, the average and beat-to-beat variability of total cosine R-to-T were not found statistically significant between both groups.

**Conclusions:**

Beat-to-beat assessment of VCG parameters may have diagnostic attributes that might help in identifying MI patients.

## Introduction

The time interval between onset of the Q-wave and offset of the T-wave, known as the QT interval, in an ECG represents the total ventricular activity of depolarization and repolarization. Elevated beat-to-beat QT interval variability (QTV) has been observed in cardiac diseases such as coronary artery disease [1], during acute myocardial ischemia [2] and before sudden cardiac death [3]. In addition, it has been shown that the elevated beat-to-beat QTV could be a marker of repolarization abnormalities in non-cardiac diseases such as obstructive sleep apnea [4] and mental disorders [5]. Moreover, it has also been proposed that the beat-to-beat QTV may be associated with the cardiac sympathetic activity [6], [7].

Generally, ECG describes the cardiac signal as amplitude but not the orientation of the heart vector direction. In addition, our recent research suggests that beat-to-beat QTV varies in inter-lead measurement, partly due to T-wave amplitude differences [8]. Vectorcardiography (VCG) is the methodological elaboration of the ECG, which measures the cardiac electrical field with both the magnitude and vector direction. More precisely, VCG aims at an orthogonal representation that reflects the electrical activity in the three perpendicular directions X, Y, and Z. Even though, the 12-lead ECG analysis is the reference setup for diagnostic purposes, the VCG is a useful alternative [9], [10], [11], [12] that represents the spatial and temporal information of cardiac activity [13], [14], [15]. Several techniques have been suggested for the derivation of VCG from standard 12-lead ECG for analysis of the cardiac signal [16], [17], [18]. Vectorcardiographic QRS-loop and T-loop analysis was demonstrated to improve risk stratification in patients with heart disease by considering a single beat of the ECG [19], [20], [21], [22].

However, the beat-to-beat variations in VCG and its descriptors are poorly understood and little is known about how these descriptors compare in different cardiac conditions. Therefore, in this study, we have investigated beat-to-beat VCG by quantifying different descriptors from the QRS and T-loop in patients with myocardial infarction (MI) as well as healthy subjects. We hypothesize that beat-to-beat variability VCG parameters may provide diagnostic information for discriminating between control subjects and MI patients.

**Figure 1:**
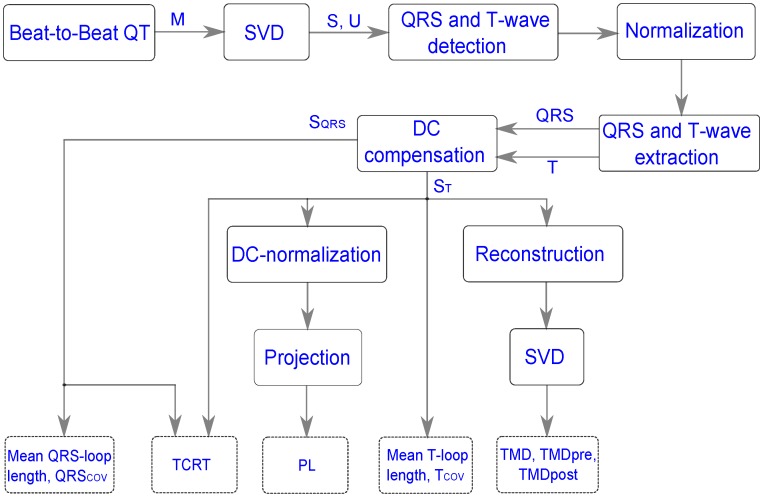
Block diagram of the overall beat-to-beat VCG approach. The solid line of rectangular or square represents the different processes or methods. The rectangles with dashed lines (at the bottom) represent the output parameters (descriptors).

## Methods

### Subjects

Standard resting 12-lead ECGs of 84 MI patients (22 females, mean age 63 ± 12 yrs and 62 males, mean age 56 ± 10) and 69 healthy subjects (17 females, mean age 42 ± 18 yrs and 52 males, mean age 40 ± 13) were investigated in this study. Most of the MI patient recordings were carried out approximately 1–2 weeks after infarction. Concerning the location of infarction, seven were anterior, 15 were antero-lateral, 20 were antero-saptal, 20 were inferior, 12 were infero-lateral, seven were infero-postero-lateral, one was lateral and two were postero-lateral. None of the patients had bundle branch block or intra-ventricular conduction defects. The QRS widths for patients with MI were comparable to those of controls (0.084 ± 0.0130 s vs. 081 ± 0.008 sec, *p* >0.05). None of the patients had a QRS width over 0.12 s.

**Figure 2:**
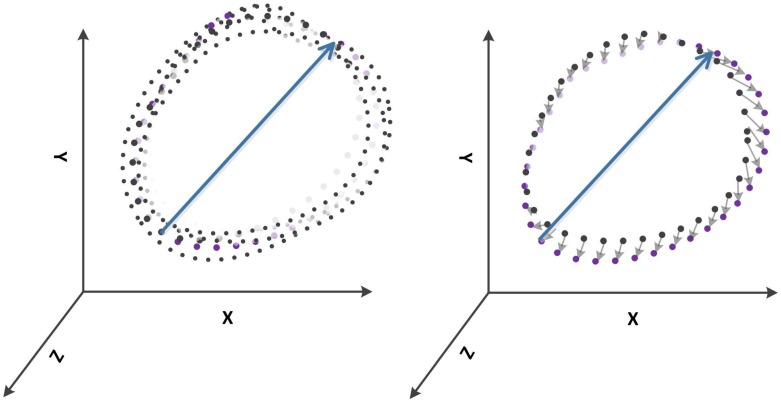
Point-to-point distance of ECG loops. The purple coloured loop depicts the mean loop. The long arrow depicts the peak of R or T-wave, respectively. Left: Schematic representation of superimposed ECG loops for several cardiac cycles. Right: The small arrows show the distance from each point of an individual loop representing one cardiac cycle to the mean loop.

The ECGs were recorded for approximately two minutes at a sampling frequency of 1000 Hz and at 16-bit resolution over a range of ± 16.384 mV. The data were obtained from the PTB diagnostic database (http://www.physionet.org/physiobank/database/ptbdb/). The data were analysed anonymously, using publicly available secondary data, therefore no ethics statement is required for this work.

**Figure 3:**
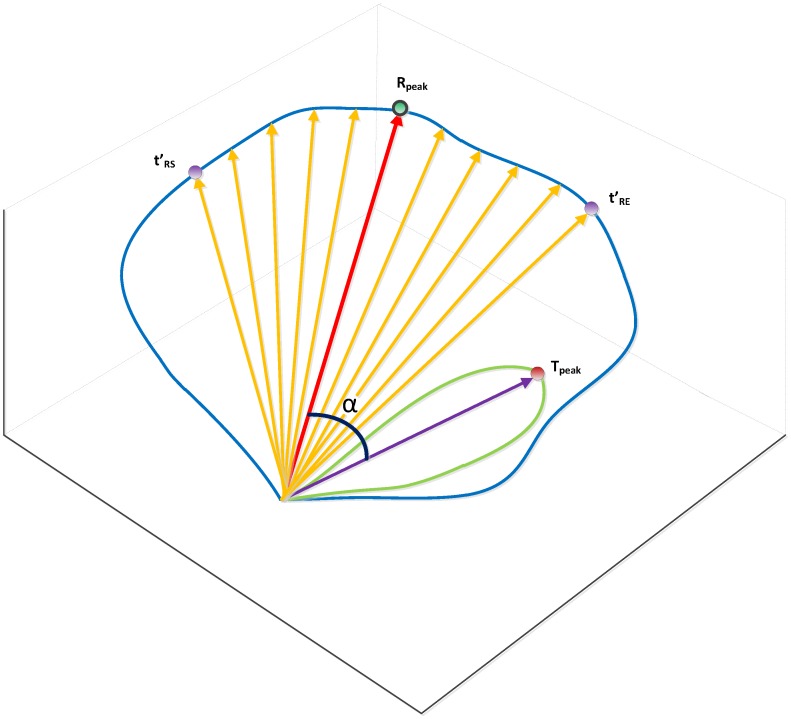
TCRT of the QRS-loop and T-loop. The ‘total cosine R-to-T’ (TCRT) describes the difference in global wavefront directions of depolarization and repolarization. The angle α is the angle between the main vectors of the QRS and T-loops.

### Spatial and Temporal VCG Analysis

The overall block diagram of ECG descriptor extraction is shown in Figure 1. To analyse spatial and temporal variations in depolarization and repolarization waves, a beat-to-beat VCG approach has been investigated in this study. The algorithm uses a robust real-time QRS detection technique that was suggested by Pan and Tompkins [23] and employs a dual-threshold approach to reduce the occurrence of false negative beats, thereby increasing the detection accuracy. If any beat is missed by the detection algorithm in one lead in a particular time instant, then a custom designed program automatically ignores the same beat of that time instant for rest of the leads. For finding the beat-to-beat QT intervals, we have used the template matching algorithm proposed by Berger and his co-workers [24]. This template matching algorithm uses an error function that was defined with respect to the sum of squared differences between the template T-wave and the stretched or compressed version of the T-wave for that beat. The algorithm finds the QT interval of all beats in a particular lead by determining how much each T-wave must be stretched or compressed in time to minimize the error function [24]. The beat-to-beat QT interval was determined for each individual lead in 12-lead ECGs by adopting an approach, which has been previously described [8]. Thereby, the beat-to-beat QRS complex onset (t'_RS_) and T**-**wave terminus locations were found. The end point of the QRS complex (t'_RE_) was obtained by adding 48 ms to the first point after the maximum of energy of the R wave falls below 70% of its maximum as originally proposed by Acar et al. [19]. The onset of the T-wave was also computed by following the approach described by Acar et al. [19]. Thus, we have found the beat-to-beat QRS and T-wave in all standard 12-lead ECGs. Ectopic beats and excessively noisy beats were automatically excluded from analysis; the latter were identified utilizing the error function of template matching.

**Table 1:** QRS and T-loop descriptors in the control and MI groups.

Descriptors	Control	MI	*p*
**Mean of DV_QRS_**	0.66±0.14	0.66±0.17	> 0.05
**SD of DV_QRS_**	0.10±0.04	0.13±0.04	< 0.0001
**Mean of DV_T_**	0.63±0.16	0.62±0.18	> 0.05
**SD of DV_T_**	0.13±0.06	0.16±0.07	< 0.05
**MLL_QRS_ (mV)**	18230±5341	14269±3507	< 0.0001
**MLL_T_ (mV)**	4332 ± 1699	3410 ± 1858	< 0.001
**Mean of TMD**	38°±16°	62°±18°	< 0.0001
**SD of TMD**	15°±6°	12°±5°	< 0.005
**Mean of TMD_pre_**	40°±18°	62°±20°	< 0.0001
**SD of TMD_pre_**	17°±6°	16°±8°	> 0.05
**Mean of TMD_post_**	34°±14°	60°±20°	< 0.0001
**SD of TMD_post_**	16°±6°	13°±5°	< 0.01
**Mean PL (%)**	55±15	46±17	< 0.001
**Mean of TRCT**	-0.04±0.43	0.03±0.45	> 0.05
**SD of TCRT**	0.12±0.07	0.13±0.12	> 0.05

**Figure 4:**
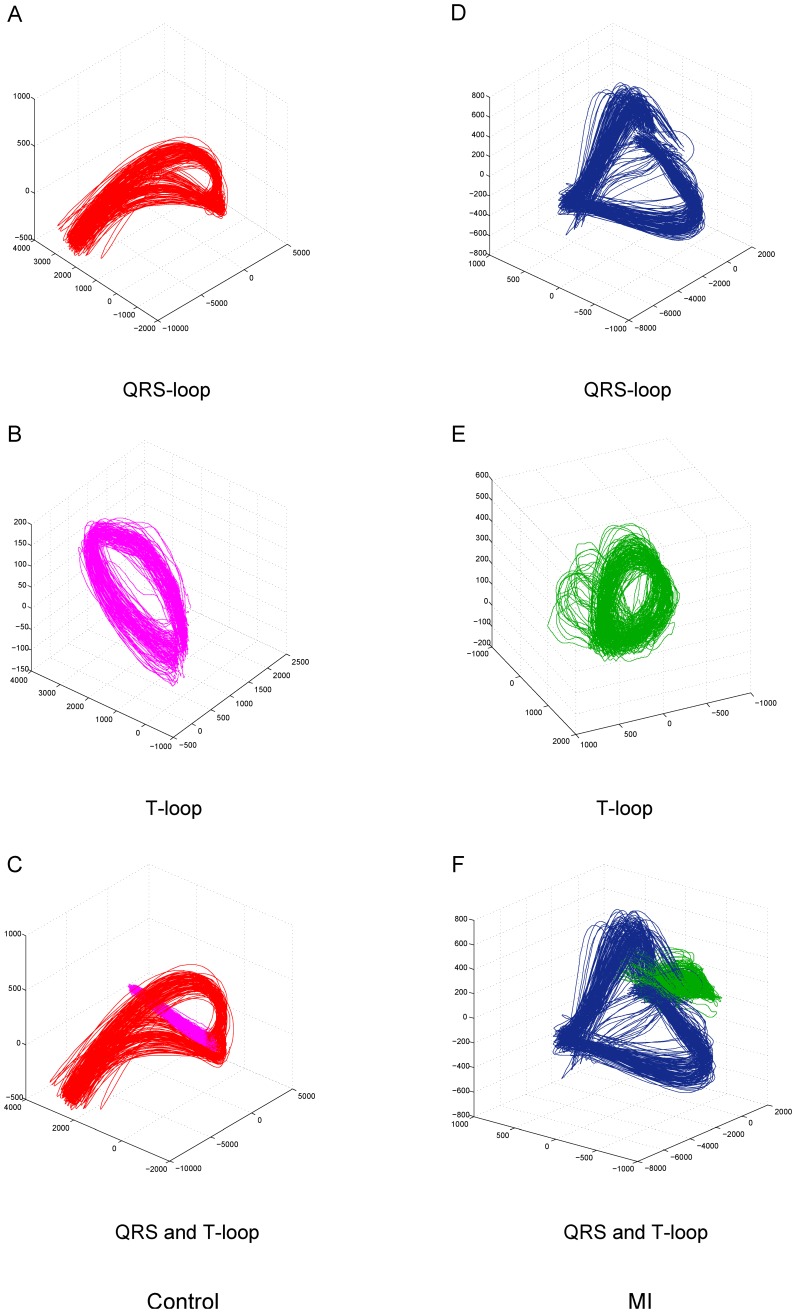
Beat-to-beat depolarization and repolarization loops in a control subject and a MI patient. The QRS (A) and T loops (B) and their combination of a normal subject are shown in red and magenta. The QRS (D) and T-loops (E) and their combination (F) of a MI patient (G) are shown in blue and green.

Descriptors of spatial and temporal wavefront characteristics for 12-lead ECG have been proposed by Acar et al. [19]. In the original and most subsequent studies only a single beat of ECG was considered. In addition, only descriptors of the T-wave morphology have been investigated. In our study, we expand this approach by developing beat-to-beat VCG analysis of ventricular depolarization and repolarization.

**Figure 5:**
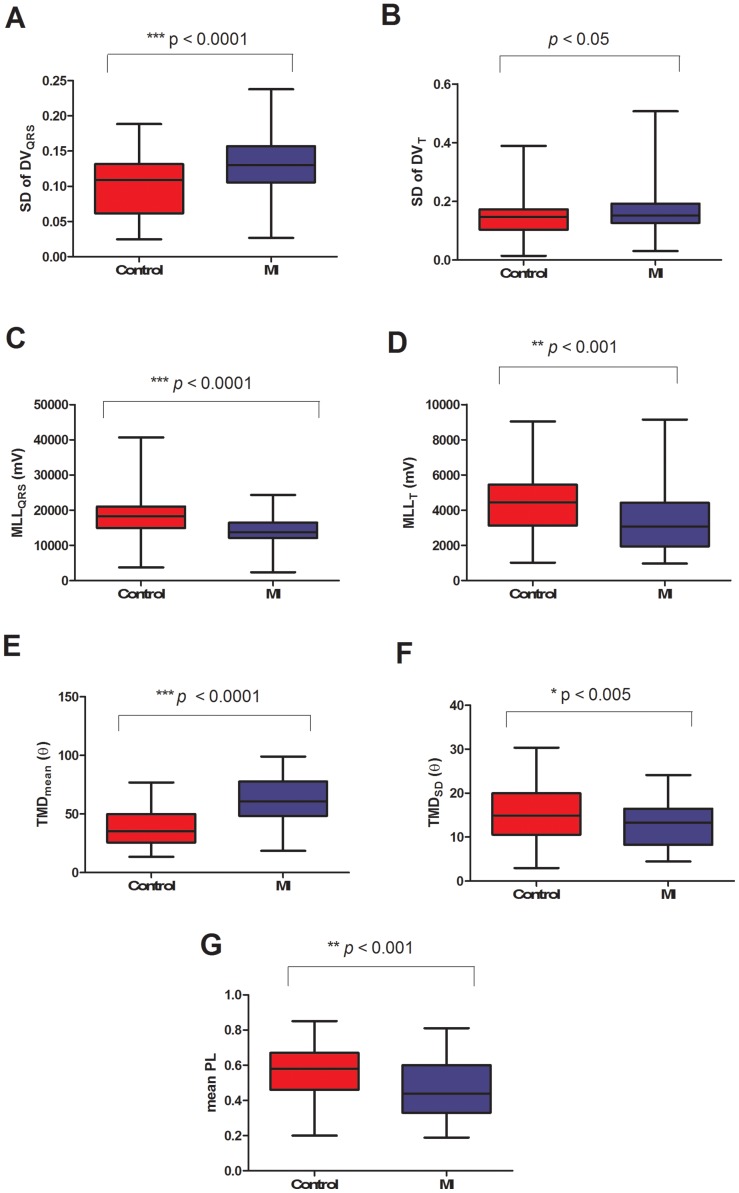
Group comparison of ventricular depolarisation and repolarization parameters. Point-to-point distance variabilityin QRS-loops (A) and T-loops (B), mean loop length of QRS-loops (C) and T-loops (D). Mean (E) and standard deviation (F) of TMD and mean PL (G) show significant differences between normal subjects and MI patients.

To derive VCG from the 12-lead ECG we employ singular value decomposition (SVD) [19]. It is assumed that **M** is an input matrix (8×*n*), where 8 represents the number of corresponding rows of ECG leads for one QT interval (I, II, V_1_–V_6_) and *n* is the number of samples. The SVD of the eight signals creates three matrices **U**, **V** and **S**, as follows.
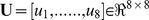


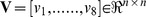


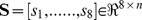


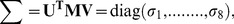
(1)where, 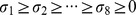 and the columns of **U** are referred to as the left singular vectors, **V** are referred to as the right singular vectors and **S** are referred to as the decomposed ECG signals for eight leads.

**Figure 6:**
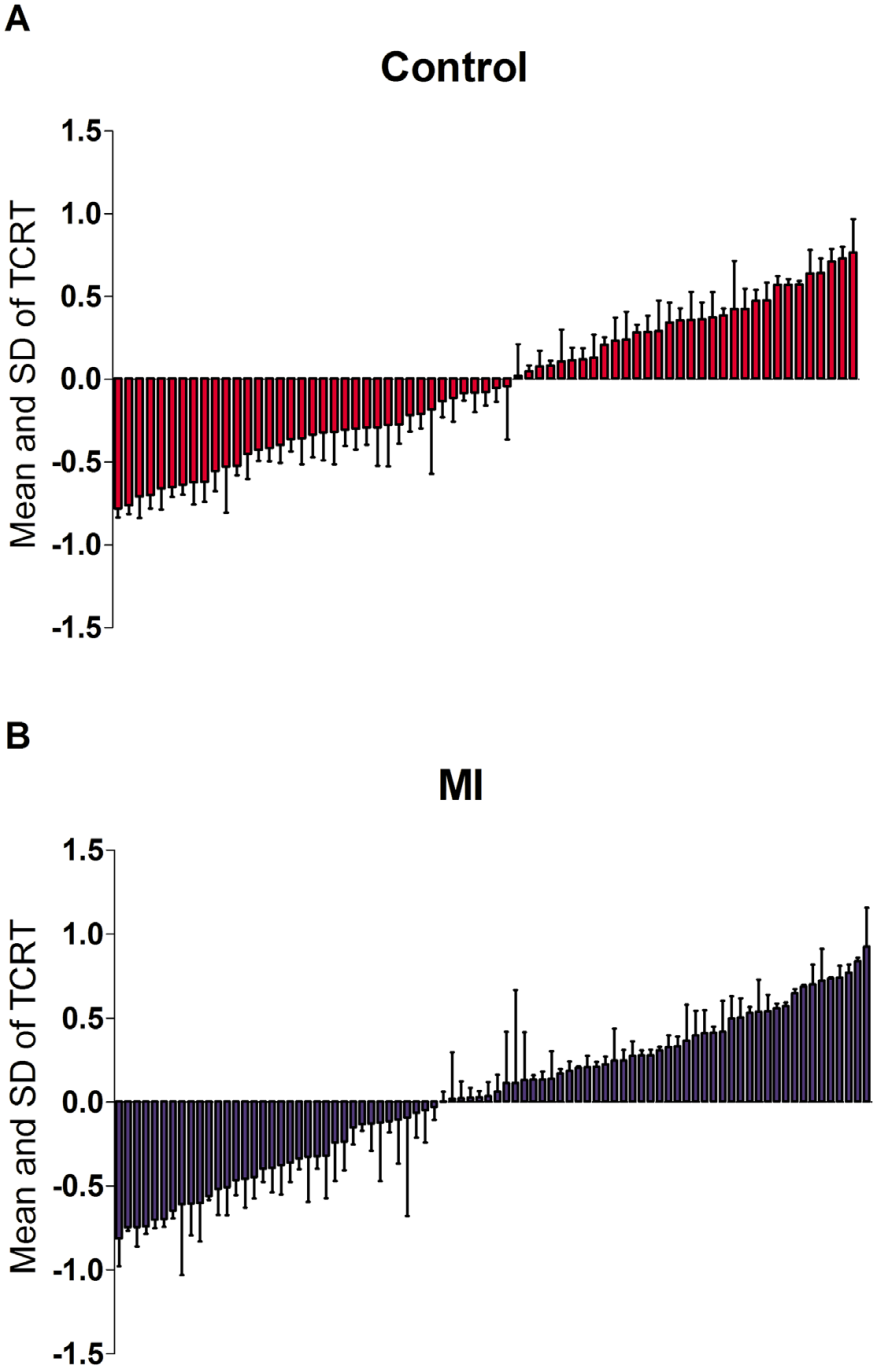
TCRT across subjects. Data are presented as mean and SD (in ascending order from left to right based on their mean value) of TCRT for individual control subjects (A) and MI patients (B).

It has been reported that 99% of the ECG energy can be represented in a three-dimensional minimum subspace [25]. Thus, the effective rank of the matrix (**M**) is three and only first three significant decomposed signals (*S_1_, S_2_, S_3_*) have been used in this study, **E**_3D_ [19], where
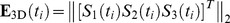
(2)


Then, the decomposed signals were normalized with the maximum energy:
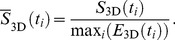
(3)


The QRS complex and T-wave signals were extracted and stored in data matrices *S*_QRS_ and *S*_T_, respectively that were proposed by [19]. The reconstructed T-wave from *S*_T_ is considered to be a form of morphological filtering.

In this study, a new parameter was computed for both QRS-complex and T-wave DV_QRS_ and DV_T_, respectively, called the point-to-point distance variability (DV). This DV was determined based on the coefficient of variance of point-to-point distance from each loop to the mean loop of ventricular depolarization and repolarization. The point-to-point distance (see Figure 2) was computed by finding the shortest distance from each point of the loops to the mean loop of QRS and T-loop, respectively. For finding the point-to-point shortest distance between loops, the k-nearest neighbours (kNN) algorithm was applied [26]. The kNN algorithm is a nonparametric method where the distance metric is calculated based on Euclidean distance. The distance between two vectors is computed as the length of the difference vector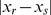, denoted by
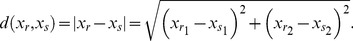
(4)


In addition, another new descriptor was computed, named the mean loop length (MLL). This MLL parameter was calculated for the mean loop of QRS and T-loop by adding the distance from each point to the next point in the loop. Moreover, for repolarization lability, three other descriptors were computed on a beat-to-beat basis, T-wave morphology dispersion TMD, TMD_pre_ and TMD_post_, as originally proposed by Acar et al. [19] for single beat ECG analysis. Further, the percentage of loop area (PL) was also determined in a beat-to-beat manner for the T**-**loop by following the same principle proposed by Acar et al. [19], but including the cells which were occupied by loop itself.To deal with beat-to-beat variability in QT intervals, which leads to beat-to-beat loops of different lengths, we truncated the QT intervals at the minimum length that is met by 90% of beats.

Finally, the relationship between the QRS and T-loops was also determined in a beat-to-beat manner through the ‘total cosine R-to-T’ (TCRT) that is the average of the cosines of the angles between the vectors of QRS (defined from t'_RS_ to t'_RE_) and the maximum of the unit vector e_T, 1_ (the vector e_T, 1_ reflects the orientation of the T-wave loop [19] as Equation 5 and shown in Figure 3).
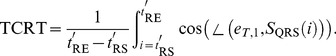
(5)


### Statistical Analysis

We have used GraphPad Prism 5^®^ (GraphPad Software, Inc., La Jolla, CA, USA), PASW Statistics 18^®^ (IBM SPSS, Inc., Somers, NY, USA) and Microsoft Excel version 2007 (Microsoft Corp., Redmond, WA, USA) for the statistical analysis. All values were expressed as mean ± standard deviation. Test results were considered statistical significant when *p* < 0.05. The beat-to-beat VCG descriptors (mean and standard deviation) were found for both the MI and the control group. Beat-to-beat variability of the VCG descriptors was calculated as standard deviation of those parameters. Further, the unpaired Student t-test was used to compare the descriptors characteristics between both studied groups.

## Results

The beat-to-beat ventricular depolarization and repolarization loops are shown in Figure 4, where the QRS-loops and T-loops are depicted separately as well as combined for both typical control subjects and MI patients, respectively. Statistical results of all descriptors are summarized in Table 1.

### Point-to-Point Distance Variability (DV) at QRS and T-loop

The mean point-to-point distance variability for QRS-loop (DV_QRS_) was not found statistically different between control and MI patients. However, the standard deviation of point-to-point distance variability in QRS loops was found to be higher in the MI group than in the control group, as shown in Figure 5A.

Similarly, the mean point-to-point distance variability of the T-loop (DV_T_) was not statistically significant. However, the standard deviation of point-to-point distance variability was significantly higher in the MI group than the control group (Figure 5B).

### Mean Loop Length (MLL)

The mean loop length of QRS-loop (MLL_QRS_) was found statistically higher in the control than the MI group (Figure 5C). Similarly, the mean loop length of T-loop (MLL_T_) was found to be higher in the control subjects than in the MI patients (Figure 5D).

### TMD, TMD_pre_andTMD_post_

The TMD parameter represents the variation of morphology of the T-wave between different ECG leads during ventricular repolarization. The mean beat-to-beat TMD was relatively lower in control subjects than MI patientsas shown in Figure 5E. However, the standard deviation of beat-to-beat TMD was significantly lower in MI patients compared to controls as shown in Figure 5F.

Looking at the T-wave morphology dispersion in more detail, we quantified TMD separately for the rising (TMD_pre_) and falling (TMD_post_) edge of the T-wave. The mean beat-to-beat TMD_pre_ was significantly higher in MI patients, but the variability of TMD_pre_ was not found to be statistically different between the control and MI group. The falling edge of mean beat-to-beat T-wave morphology dispersion was found to be lower in control subjects than in MI. Conversely, the variability of this parameter was reduced in MI patients compared to controls as shown in Table 1.

### Loop Area

The PL for mean T-wave loop was higher in the control subjects compared to MI patients as shown in Figure 5G.

### TCRT

The negativity of TCRT quantifies the deviation in the orientation of depolarization and repolarization loops. In our study, the mean TCRT showed no statistically significant difference between control subjects and MI patients. Similarly, the beat-to-beat variability of TCRT was comparable between MI patients and control subjects. Figure 6 shows the mean and SD values of TCRT for all control subjects and MI patients.

## Discussion

Our study proposes an approach for analysing the beat-to-beat spatial and temporal variations of ventricular depolarization and repolarization wavefronts and evaluates the value of those parameters for characterising cardiac electrical abnormalities in patients with MI.

The main finding of our study is an increase in variability of depolarization as well as repolarization in patients with MI compared to normal subjects. Our beat-to-beat VCG approach demonstrated increased instability in depolarization and repolarization in MI patients as demonstrated by increased point-to-point distance variability (Figure 5A and 5B). This is in line with previous observations of increased QRS [27] and QT variability [28] observed in the 12 lead ECG in patients with coronary artery disease and following myocardial infarction. Both, QRS and T-loops were significantly shorter in MI patients compared to controls (Figure 5C and 5D) and suggest less coordinated conduction and repolarization of the ventricular myocardium, which would result in smaller magnitudes of QRS and T vectors.

Along with our new parameters, some other parameters were also investigated in a beat-to-beat manner to quantify the discriminative power. Our observations confirm that MI patients have higher TMD values compared to the normal subjects [29], which indicates that the reconstruction vectors for control subjects were closely grouped (i.e. have similar morphology) and relatively dispersed in the MI patients. Another study showed significant predictive value of TMD for cardiovascular mortality in the general male population [30]. The beat to-beat variability of TMD was found decreased in patients with MI. However, our study suggests that the group difference in the average value of TMD is more significant than its beat-to-beat variability.

Further, the investigation of TMD by considering TMD_pre_ (rising edge of T-wave) and TMD_post_(falling edge of T-wave) demonstrated that dispersion was in both parts of the T-wave higher in the MI than in the control subjects. However, it appears that the last part of the T-wave might be more indicative of repolarization abnormalities in MI patients and is in line with reports on the prognostic value of the T_peak_-T_end_ interval of ECG [31], [32]. However, the beat-to-beat variability of these parameters shows a different scenario. No significant difference between control and MI group in the variability of TMD_pre_ was observed, but a significant decrease in the variability of TMD_post_ was found in MI patients. The cause for reduced beat-to-beat variability in TMD_post_ of MI patients is not clear.

The TCRT descriptorthat measures the vector deviation between the depolarization and repolarization waves has been investigated in several studies [19], [33] and was found to be an independent predictor of cardiac mortality in some studies [30], [34], but not in others [35], [36]. However, the majority of previous studies were limited to single analysis of beat of QRS and T-loop. Our study did not show a significant difference in the mean value of TCRT between MI patients and normal subjects. Beat-to-beat analysis of TCRT in our study demonstratedcomparable variability in MI patients and controls and is in contrast with other studies, which suggest that TCRT might have prognostic value when analysed in a beat-to-beat manner [37], [38]. Importantly, the aforementioned studies investigated TCRT variability during exercise tests.

In addition, we have observed that control subjects have higher values of PL than MI patients, which indicates relatively smooth and connected average T-loops in control subjects compared to MI patients, further emphasising the presence of repolarization abnormalities.

In summary, significant changes in ventricular conduction and repolarization processes post MI could be detected in a variety of VCG parameters, capturing features of the averaged cardiac cycle and beat-to-beat lability.

### Limitations

The information on the subjects included in the PTB database is limited and, for example, does not contain BMI values for both MI patients and healthy subjects. Also, the average age in the MI group was significantly higher than in the control group and might have had an impact on our results. In addition, the duration of the ECG recordings for this study was relatively short and longer duration of data could increase the statistical performance of the vectorcardiographic QRS and T-loop descriptors. Further, we did not compare the performance of our VCG approach to more established techniques, e.g. single lead QT variability assessment. The method of QRS end estimation that was employed in this study might be a considerable limitation if patients with ventricular conduction abnormalities were to be considered. However, in this study, none of the ECG had a QRS width over 0.12 s.

### Conclusion

Vectorcardiographic analysis of beat-to-beat variability in ventricular depolarization and repolarization may provide markers of electrical instability in the heart of patients with myocardial infarction.
